# Resistance training increases muscle NAD^+^ and NADH concentrations as well as NAMPT protein levels and global sirtuin activity in middle-aged, overweight, untrained individuals

**DOI:** 10.18632/aging.103218

**Published:** 2020-05-05

**Authors:** Donald A. Lamb, Johnathon H. Moore, Paulo Henrique Caldeira Mesquita, Morgan A. Smith, Christopher G. Vann, Shelby C. Osburn, Carlton D. Fox, Hector L. Lopez, Tim N. Ziegenfuss, Kevin W. Huggins, Michael D. Goodlett, Andrew D. Fruge, Andreas N. Kavazis, Kaelin C. Young, Michael D. Roberts

**Affiliations:** 1 Department of Nutrition, Dietetics and Hospitality Management, Auburn University, Auburn, AL 36849, USA; 2 School of Kinesiology, Auburn University, Auburn, AL 36849, USA; 3 Center for Applied Health Sciences, Canfield, OH 44406, USA; 4 Athletics Department, Auburn University, Auburn, AL 36849, USA; 5 Edward Via College of Osteopathic Medicine, Auburn, AL 36832, USA

**Keywords:** muscle, resistance training, aging, NAD ^+^, NADH

## Abstract

We examined if resistance training affected muscle NAD^+^ and NADH concentrations as well as nicotinamide phosphoribosyltransferase (NAMPT) protein levels and sirtuin (SIRT) activity markers in middle-aged, untrained (MA) individuals. MA participants (59±4 years old; n=16) completed 10 weeks of full-body resistance training (2 d/wk). Body composition, knee extensor strength, and vastus lateralis muscle biopsies were obtained prior to training (Pre) and 72 hours following the last training bout (Post). Data from trained college-aged men (22±3 years old, training age: 6±2 years old; n=15) were also obtained for comparative purposes. Muscle NAD^+^ (+127%, p<0.001), NADH (+99%, p=0.002), global SIRT activity (+13%, p=0.036), and NAMPT protein (+15%, p=0.014) increased from Pre to Post in MA participants. Additionally, Pre muscle NAD^+^ and NADH in MA participants were lower than college-aged participants (p<0.05), whereas Post values were similar between cohorts (p>0.10). Interestingly, muscle citrate synthase activity levels (i.e., mitochondrial density) increased in MA participants from Pre to Post (+183%, p<0.001), and this increase was significantly associated with increases in muscle NAD^+^ (r^2^=0.592, p=0.001). In summary, muscle NAD^+^, NADH, and global SIRT activity are positively affected by resistance training in middle-aged, untrained individuals. Whether these adaptations facilitated mitochondrial biogenesis remains to be determined.

## INTRODUCTION

Nicotinamide adenine dinucleotide (NAD^+^) is a metabolite involved in numerous biochemical reactions. In particular, NAD^+^ is involved with electron transport where the reduced form (NADH) transfers electrons to other substrates and intermediates of metabolism. There is enthusiasm surrounding the role that tissue NAD^+^ concentrations play in the aging process (reviewed in [1–3]), and researchers have determined skeletal muscle NAD^+^ concentrations are lower in older rodents and humans [4–6]. These findings have led some to suggest that the age-associated loss in skeletal muscle NAD^+^ levels contributes to mitochondrial dysfunction (reviewed in [7]). Multiple enzymes act to catalyze the formation of NAD^+^ from the amino acid tryptophan via the kynurenine *de novo* pathway [8]. Additionally, NAD^+^ biosynthesis can be catalyzed through the salvage/recycling pathway, and nicotinamide phosphoribosyltransferase (NAMPT) is the rate-limiting enzyme in this pathway [9]. NAMPT has been shown to play a critical role in muscle cell differentiation, metabolism and senescence *in vitro* [10]. Additionally, there is evidence to suggest skeletal muscle NAMPT protein levels are lower in older versus younger humans [11], and this finding re-iterates the notion that maintaining skeletal muscle NAD^+^ concentrations may be integral in maintaining metabolic homeostasis.

Beyond its involvement with redox reactions, NAD^+^ binds to and activates a class of enzymes that possess deacetylase activity called sirtuins (SIRTs) [12, 13]. While there are seven SIRT enzymes, SIRT1 and SIRT3 play prominent roles in skeletal muscle mitochondrial metabolism. SIRT1 is a nuclear NAD^+^-dependent deacetylase that links cell metabolism to transcriptional regulation. For example, SIRT1 acts as a transcriptional regulator for the peroxisome proliferator activated receptor-γ co-activator-1α (PGC-1α) gene [14], which has been deemed as a nodal regulator of mitochondrial biogenesis (reviewed in [15]). There is additional evidence suggesting SIRT1 can deacetylate the PGC-1α protein in skeletal muscle which, in turn, leads to an increase in PGC-1α activity and mitochondrial fatty acid oxidation [16]. Conversely, SIRT3 influences metabolism by deacetylating and activating mitochondrial enzymes such as pyruvate dehydrogenase and long-chain acyl coenzyme A dehydrogenase [17, 18]. Thus, an age-related loss in skeletal muscle NAD^+^ concentrations likely leads to a decrease in SIRT1/3 activities, which may then contribute to mitochondrial dysfunction and muscle aging.

Endurance training appears to be capable of increasing skeletal muscle markers related to NAD^+^ and SIRT signaling. For instance, endurance training in rodents and humans has been shown to modulate SIRT1 and SIRT3 protein levels and increase the activity of these enzymes in skeletal muscle [5, 19, 20]. Additionally, skeletal muscle NAMPT protein levels have been reported to be higher in endurance-trained athletes versus untrained individuals [21]. However, there is a paucity of research examining these biomarkers in response to resistance training. It remains plausible that resistance training can increase skeletal muscle markers related to NAD^+^ biosynthesis and SIRT signaling, and this may be an involved mechanism in facilitating training adaptations. Given the paucity of data in this area, we sought to examine the effects of resistance training on skeletal muscle NAD^+^ concentrations as well as NAMPT protein levels, SIRT1/3 protein levels, and markers of SIRT activity in middle-aged, overweight, untrained individuals. We also sought to compare assayed biomarkers from these middle-aged individuals to a cohort of recreationally trained college-aged individuals to determine if training was capable of potentially restoring these markers to levels observed in recreationally trained college-aged males. Finally, we examined muscle citrate synthase activity levels in the middle-aged participants to determine if: i) training increased this marker (suggestive of increased mitochondrial density), and/or ii) training-induced changes in muscle NAD^+^ or NADH concentrations were associated with training-induced changes in this marker.

## RESULTS

### Participant characteristics and resistance training adaptations in middle-aged, untrained participants

Middle-aged participants (referred to as ‘MA’ in the results and figures; n=16, n=6 males and 10 females) were 59±4 years of age, and (prior to training) possessed a body mass index (BMI) of 31.7±5.6 kg/m^2^, possessed a fat-free mass index (FFMi; DXA FFM in kg divided by height in m^2^) of 18.0±2.9 kg/m^2^, and possessed a body fat percentage of 39.3±6.3%. Recreationally-trained, college-aged participants (n=15 males) were 22±3 years old, self-reported resistance training for 6±2 years (range: 4-10 years), possessed a BMI of 26.7±3.3 kg/m^2^, possessed a FFMi of 20.6±2.0 kg/m^2^, and possessed a body fat percentage of 19.0±6.5%. There were significant differences between cohorts for age (p<0.001), BMI (p=0.006), FFMi (p=0.009), and body fat percentage (p<0.001).

Table 1 contains Pre and Post variables indicative of general resistance training adaptations for the MA group. While there was not a significant change in whole-body DXA FFM (+1.0±1.9 kg, p=0.061), vastus lateralis thickness as well as knee extensor peak torque significantly increased with training (p=0.038 and p=0.031, respectively). In the MA group, all three variables at Pre and Post were significantly lower compared to the college-aged group (p<0.05). In MA participants, no significant gender×time interactions existed for Pre to Post changes in DXA FFM (p=0.282), vastus lateralis thickness (p=0.634), or knee extensor peak torque (p=0.916).

**Table 1:** Phenotype data.

**Variable (units)**	**Mean ± SD**	
DXA Fat-free mass (kg)	Middle-aged	
	Pre	53.9±10.5^#^
	Post	54.9±10.4^#^
	Younger, trained	69.2±7.7
VL muscle thickness (cm)	Middle-aged	
	Pre	1.88±0.45^#^
	Post	2.02±0.37^*,#^
	Younger, trained	2.87±0.35
VL peak knee extensor torque (N•m)	Middle-aged	
	Pre	115±43^#^
	Post	127±40^*,#^
	Younger, trained	230±46

Legend: Data are from n=16 middle-aged participants prior to and following 10 weeks of training as well as basal values in n=15 college-aged participants that were recreationally trained. Abbreviations: DXA, dual x-ray absorptiometry; VL, vastus lateralis. Symbols: *, indicates increase from Pre to Post in middle-aged participants (p<0.05); #, indicates different from younger, trained participants (p<0.05).

### Muscle NAD^+^, NADH, and NAMPT protein levels

**Figure 1:**
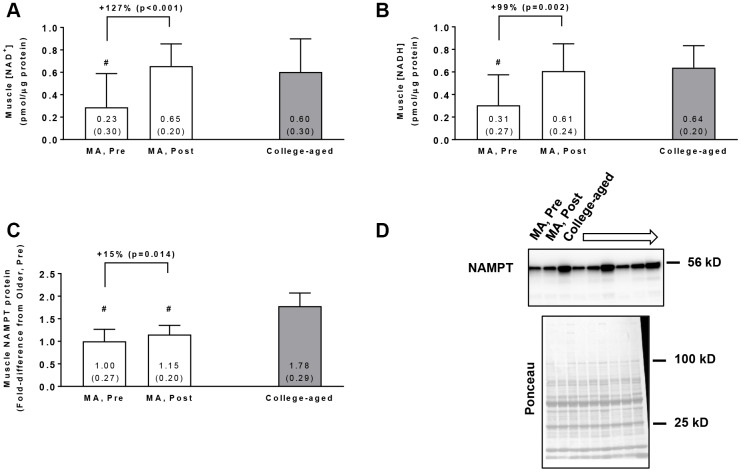
Vastus lateralis muscle tissue NAD^+^ concentrations (**A**), NADH concentrations (**B**), and NAMPT protein levels (**C**) in middle-aged (MA) participants prior to (Pre) and following 10 weeks of resistance training (Post) as well as basal values in college-aged participants that were recreationally trained. All data are presented as means±SD values. Panel (**D**) shows a representative Western blot for n=3 middle-aged participants (Pre and Post training) as well as n=3 college-aged participants. All middle-aged participants were assayed for NAMPT; however, 15/16 middle-aged and 14/15 college-aged participants were assayed for muscle NAD^+^ and NADH concentrations due to tissue limitations. Symbols: #, indicates value in older group at Pre and/or Post training was less than college-aged cohort (p<0.05).

In the MA group, training increased muscle NAD^+^ concentrations (p<0.001, Figure 1A) and muscle NADH concentrations (p=0.002, Figure 1B). Additionally, Pre NAD^+^ and NADH concentrations in MA participants were lower compared to the college-aged cohort (p=0.009 and p<0.001, respectively), whereas Post values were similar to the college-aged cohort (p=0.603 and p=0.775, respectively). In the MA group, training also increased muscle NAMPT protein levels (p=0.014, Figure 1C), although levels at Pre and Post were lower than the college-aged cohort (p<0.001). In MA participants, no significant gender×time interactions existed for Pre to Post changes in muscle NAD^+^ concentrations (p=0.099), muscle NADH concentrations (p=0.617), or muscle NAMPT protein levels (p=0.143).

### Muscle SIRT activity markers

**Figure 2:**
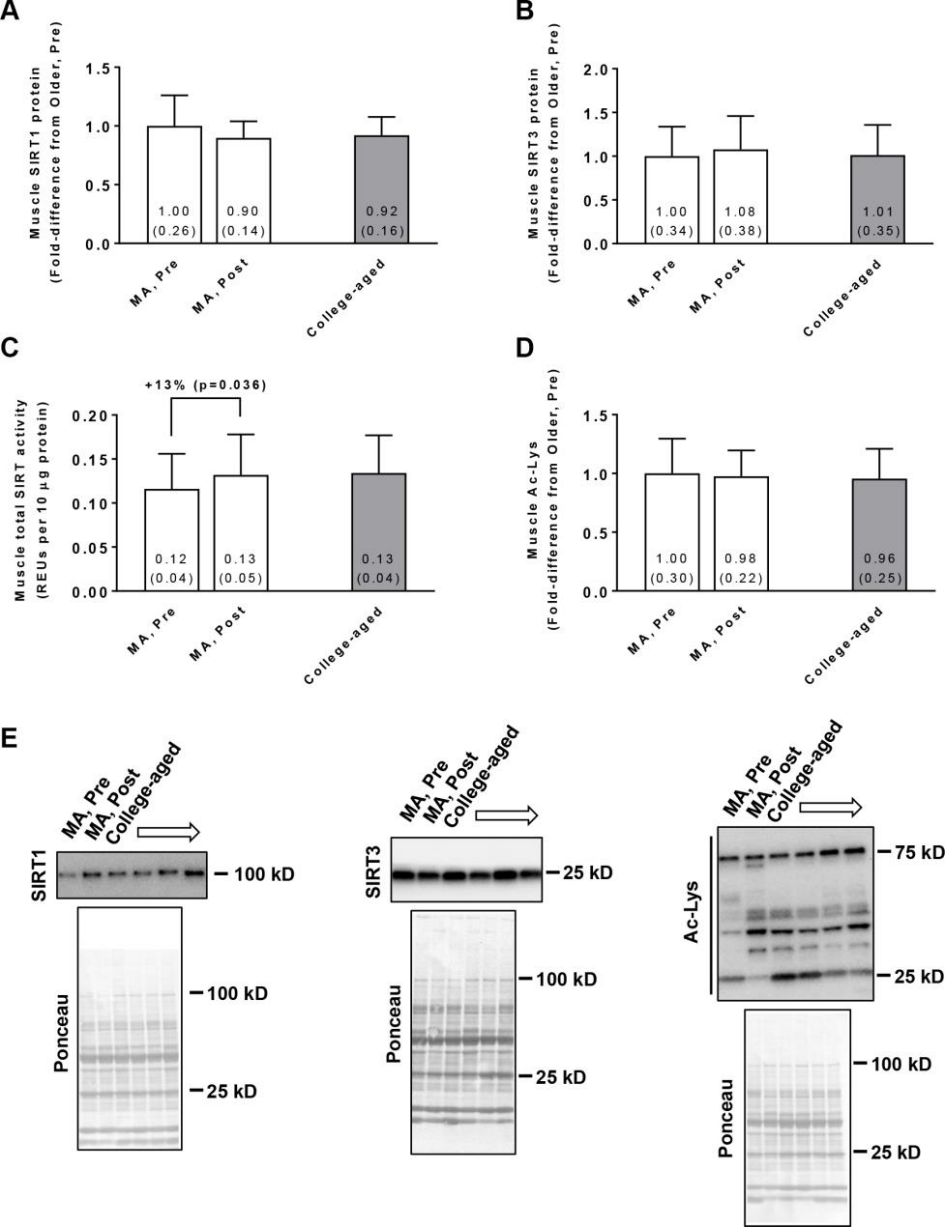
Vastus lateralis muscle tissue SIRT1 protein levels (**A**), SIRT3 protein levels (**B**), global sirtuin (SIRT) activity (**C**), and acetylated protein levels (**D**) in middle-aged (MA) participants prior to (Pre) and following 10 weeks of resistance training (Post) as well as basal values in college-aged participants that were recreationally trained. All data are presented as means±SD values. Panel (**E**) shows representative Western blots for n=2 middle-aged participants (pre and Post) as well as n=2 college-aged participants. All middle-aged participants were assayed; however, 14/15 college-aged participants were assayed due to tissue limitations.

In the MA group, training did not affect muscle SIRT1 protein levels (Pre → Post p=0.210; Figure 2A) or SIRT3 protein levels (Pre → Post p=0.181; Figure 2B). Training did, however, increase global SIRT activity in these participants (Pre → Post p=0.036; Figure 2C). Training in MA participants did not affect acetylated protein levels (Pre → Post p=0.519; Figure 2D). At Pre or Post, values for these variables were similar between MA and college-aged participants. In MA participants, no significant gender×time interactions existed for Pre to Post changes in muscle SIRT1 protein levels (p=0.926), muscle SIRT3 protein levels (p=0.764), muscle SIRT activity levels (p=0.481), or muscle acetylated protein levels (p=0.496).

### Muscle citrate synthase activity levels in middle-aged participants at Pre and Post

**Figure 3:**
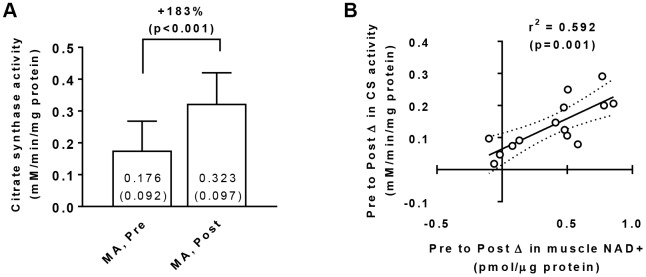
Vastus lateralis muscle tissue citrate synthase activity levels in middle-aged (MA) participants prior to (Pre) and following 10 weeks of resistance training (Post) (**A**), as well as associations between the Pre-to-Post changes in this variable and muscle NAD^+^ concentrations (**B**). Data in panel (**A**) are presented as means±SD values. Data in panel (**B**) are presented as individual data points where the regression line is solid and 95% confidence interval bands are dashed. Only 15/16 middle-aged participants were assayed for citrate synthase activity levels at Pre and Post due to tissue limitations for one participant.

Training increased muscle citrate synthase activity levels in MA participants (Pre → Post p<0.001; Figure 3A) suggestive of increased mitochondrial density, and a significant gender×time interaction did not exist for Pre to Post changes in this marker (p=0.070). Interestingly, there was a significant association between training-induced changes in muscle citrate synthase activity levels and changes in muscle NAD^+^ concentrations (r^2^=0.592, p=0.001; Figure 3B). There was also a significant association between training-induced changes in muscle citrate synthase activity levels and changes in muscle NADH concentrations (r^2^=0.413, p=0.013; not in figure).

## DISCUSSION

We sought to examine if resistance training affected skeletal muscle NAD^+^ and NADH concentrations as well as NAMPT protein levels, SIRT1/3 protein levels, and markers of SIRT activity in middle-aged, overweight, previously untrained individuals. We also sought to compare the Pre and Post-training values in these participants to recreationally trained college-aged participants. Finally, we sought to determine if resistance training increased muscle citrate synthase activity levels in the middle-aged participants, and determine whether changes in this marker were associated with changes in muscle NAD^+^ levels. In the middle-aged participants, the 10-week training intervention: i) promoted training adaptations (i.e., increased strength and localized hypertrophy), ii) robustly increased muscle NAD^+^ and NADH concentrations, iii) modestly (but significantly) increased NAMPT protein levels and global SIRT activity, and iv) robustly increased citrate synthase activity levels in muscle suggesting mitochondrial biogenesis occurred. Muscle SIRT1/3 protein levels as well as acetylated lysine levels were not affected with training in the middle-aged participants, and were not different between the middle-aged and college-aged cohorts.

SIRTs are activated by NAD^+^, and these enzymes are intimately involved with skeletal muscle metabolism. As mentioned prior, endurance training in humans and rodents has been shown to modulate SIRT1 and SIRT3 protein levels and increase the activity of these enzymes in skeletal muscle [5, 19, 20]. Additionally, skeletal muscle NAMPT protein levels have been reported to be higher in endurance-trained versus sedentary individuals [21]. Notwithstanding, human studies examining these biomarkers in response to resistance training are lacking, and only two studies have examined how resistance training affects skeletal muscle NAMPT protein levels in humans. The first study, performed in college-aged participants, indicated that three weeks of leg extensor training increased skeletal muscle NAMPT protein levels by 16% [22]. The second study, performed in college-aged and older individuals, indicated that 12 weeks of resistance training increased skeletal muscle NAMPT protein expression in both age groups [11]. While insightful, neither study examined muscle NAD^+^/NADH concentrations or SIRT activity markers. The current study agrees with these prior findings regarding NAMPT protein changes with resistance training, and extend upon these findings by suggesting resistance training increases muscle NAD^+^ and NADH concentrations as well as global SIRT activity. We posit that the training-induced increase in muscle NAD^+^ and NADH concentrations was likely facilitated through increased NAMPT protein levels since this is the rate-limiting enzyme in the intracellular salvage of NAD^+^ from NAM. It is also likely that increased muscle NAD^+^ concentrations, rather than increases in SIRT enzyme levels per our Western blotting results, contributed to increased SIRT activity.

Given that increased NAMPT protein levels may be the primary mechanism through which increased net NAD^+^/NADH concentrations and global SIRT activity occurred, determining how resistance training affects NAMPT mRNA and protein expression is warranted. Prior literature has established the 5' AMP-activated protein kinase (AMPK) enzyme transcriptionally controls NAMPT expression in skeletal muscle [22], and it has been demonstrated that an acute bout of resistance training increases markers of AMPK activity [23–25]. Thus, pulsatile increases in muscle AMPK activity during bouts of training may be an impetus to stimulate *NAMPT* mRNA expression, and these events may eventually lead to an increased accretion of NAMPT protein with longer-term training. However, *NAMPT* has also been shown to be transcriptionally regulated by circadian transcription factors [26] and JAK-STAT signaling [27]. Thus, it remains unclear how resistance training increases muscle NAMPT protein levels, and this should be further elucidated.

Other intriguing findings herein included the robust training-induced increases in muscle citrate synthase activity levels in the middle-aged participants as well as the significant association between changes in this marker with changes in muscle NAD^+^ levels. The former finding suggests resistance training likely increased mitochondrial density through increased biogenesis in skeletal muscle, and the latter finding (while only an association) may suggest that increased NAD^+^ levels could be a mechanistic driver of this phenomenon. The training literature is mixed with regard to how resistance training affects mitochondrial density. To this end, several resistance training studies have reported that mitochondrial density remains unaltered [28–30] or decreases [31–35], and fewer studies have shown resistance training increases mitochondrial density [36]. We recently posited that, while most of the literature indicates mitochondrial density is not affected in younger individuals, resistance training may facilitate this adaption in older and more sedentary individuals due to increased metabolic demand [37]. However, we did not previously speculate that resistance training-induced increases in muscle NAD^+^ concentrations were responsible for this adaptation. Given the compelling association reported herein, as well as the relationship between NAD^+^ and mitochondrial biogenesis [38, 39], more research should be performed to validate this potential mechanism.

It is also noteworthy that, while exercise is capable of increasing muscle NAD^+^ levels, nicotinamide riboside (NR) supplementation with the intent of increasing blood and tissue NAD^+^ levels has gained recent notoriety [40, 41]. The overall health benefits of such supplementation strategies remain unknown. However, recent data are emerging in this area of human physiology. For instance, while NR supplementation has not been shown to affect skeletal muscle mitochondrial physiology in obese and insulin-resistant men [42], another recent study determined NR supplementation reduced circulating levels of inflammatory cytokines [43]. Determining whether exercise training-induced increases in NAD^+^ biosynthesis versus NR supplementation-induced increases in tissue NAD^+^ levels produce similar outcomes is warranted.

### Experimental considerations

One notable limitation is the short-term duration of training in middle-aged participants. Thus, longer-term training studies should be pursued to determine if similar outcomes are observed. Another limitation includes the utilization of recreationally trained men as the younger comparator group. This is an unresolved limitation, and future studies are needed to examine the biomarkers interrogated herein between younger and older males and females who are exercise trained and untrained. Obtaining such data will provide a clearer picture as to whether muscle NAD^+^, NADH, and SIRT markers associate with various muscle phenotypes. Global acetylated lysine levels remained unaltered with training, and were not different between age cohorts. This finding was likely due to our interrogation of whole tissue lysates rather than examining subcellular fractions (e.g., nuclear and/or mitochondrial extracts); notably, we were limited in performing subcellular fractionation due to low biopsy yields. Prominent sites of SIRT activity are in the nucleus and the mitochondria. For instance, mitochondrial proteins have been shown to be de-acetylated by SIRT3 in rodents [44], and nuclear histones have been shown to be de-acetylated by SIRT6 in yeast [45]. Thus, our SIRT data are also limited to whole tissue lysates, and future studies should aim to fractionate biopsy tissue prior to examining these biomarkers. Finally, one issue not clarified with the current dataset is the putative benefit of increased muscle NAD^+^, NADH, and SIRT activity with resistance training. Indeed, the association between training-induced increases in muscle citrate synthase activity and NAD^+^ levels is intriguing. As mentioned throughout, however, there are data which also link increased SIRT activity to enhanced mitochondrial function. In this regard, our current data are limited given that markers of mitochondrial function (e.g., respiratory markers and complex activities) were not examined herein. Certain studies have shown that, in older individuals, resistance training increases mitochondrial complex activity [29], state III respiration [46], and markers of mitochondrial biogenesis [47]. However, whether the aforementioned phenomena were due to increases in muscle NAD^+^ or NADH concentrations remains to be determined.

## CONCLUSIONS

This is the first evidence to suggest resistance training in middle-aged individuals restores muscle NAD^+^ and NADH concentrations to levels observed in recreationally-trained college-aged individuals. The finding of increased global SIRT activity is also novel, and the current data confirm that resistance training is capable of increasing muscle NAMPT protein levels. Finally, the training-induced increases in muscle mitochondrial density along with the association between increases in muscle citrate synthase activity and NAD^+^ levels are intriguing and warrant further investigation.

## MATERIALS AND METHODS

### Ethical approval

This study was a secondary analysis of two studies approved by the Institutional Review Board at Auburn University. The first protocol (Protocol # 19-249 MR 1907) involved examining the effects of resistance training with daily peanut protein supplementation or no supplementation on skeletal muscle hypertrophy in middle-aged, untrained males and females aged 50 years and older (NCT04015479). While subjects from both groups were examined herein, two-way repeated measures ANOVAs indicated that none of the assayed biomarkers were affected by supplementation (interaction p>0.50 for all assayed variables). The second protocol involved examining the effects of unilateral resistance training on muscle hypertrophy outcomes in previously trained college-aged males (Protocol # 19-245 MR 1907).

Inclusion criteria for both studies required participants to abstain from nutritional supplementation one month prior to testing. Participants from both studies had to be free of overt cardio-metabolic diseases (e.g., type II diabetes, severe hypertension, heart failure) or conditions that precluded the collection of a skeletal muscle biopsy. All participants provided verbal and written consent to participate in each respective study, and both studies conformed to standards set by the latest revision of the Declaration of Helsinki.

### Testing sessions

For middle-aged participants, the testing sessions described below occurred during morning hours (05:00–09:00) following an overnight fast for all but 3 participants who reported to the laboratory after working hours at 17:00-18:30 following a ~4-5 hour fast. For all participants, Pre testing occurred ~2-5 days prior to the first day of resistance training, and Post training occurred 72 hours following the last training bout. The younger participants performed all of the same tests described above between 05:00 and 11:00, and this testing session occurred 10 days following their last lower-body training bout.

Prior to testing batteries, participants submitted a urine sample (~5 mL) to assess urine specific gravity (USG) levels using a handheld refractometer (ATAGO; Bellevue, WA, USA). Notably, USG levels in all participants were <1.020. Height and body mass were assessed using a digital column scale (Seca 769; Hanover, MD, USA) with weights and heights being collected to the nearest 0.1 kg and 0.5 cm, respectively. Participants then partook in a full body dual x-ray absorptiometry (DXA) scan in order to determine bone-free fat-free mass (FFM) and fat mass (Lunar Prodigy; GE Corporation, Fairfield, CT, USA). The same investigator completed all DXA scans. According to previous data published by our laboratory [48], the same-day test-calibrate-retest reliability on 10 participants produced an intra-class correlation coefficient (ICC) of 0.998 for FFM.

Right leg vastus lateralis ultrasound assessments were performed using a 3-12 MHz multi-frequency linear phase array transducer (Logiq S7 R2 Expert; General Electric, Fairfield, CT, USA) to determine muscle thickness as previously described by our laboratory [49, 50]. Participants were instructed to stand and displace bodyweight more to the left leg to ensure the right leg was relaxed. Measurements were standardized by placing the transducer at the midway point between the iliac crest and patella. The same technician performed all ultrasounds. According to previous data published by our laboratory [51], the same-day test-retest reliability for muscle thickness assessment on 30 participants produced an ICC of 0.99.

Right leg vastus lateralis muscle biopsies were obtained with a 5-gauge needle as previously described by our laboratory [52]. Following biopsies, tissue was rapidly teased of blood and connective tissue, and subsequently stored at -80°C for further molecular analyses.

In middle-aged participants, right leg extensor peak torque testing occurred ~1-3 days prior to the muscle biopsy at the Pre time point, whereas this test occurred approximately 10 minutes following the biopsy at the Post test. This difference in methodology between time points was due to logistical constraints. However, we have unpublished data suggesting peak torque values are not affected by muscle biopsies when isokinetic testing occurs within a 10-minute post-biopsy window. In younger participants, this test occurred approximately 10 minutes following the biopsy. During testing participants were fastened to an isokinetic dynamometer (Biodex System 4, Biodex Medical Systems, Inc., Shirley, NY, USA). Each participant’s knee was aligned with the axis of the dynamometer, and seat height was adjusted to ensure the hip angle was approximately 90°. Prior to peak torque assessment, each participant performed a warmup consisting of submaximal to maximal isokinetic knee extensions. Participants then completed five maximal voluntary isokinetic knee extension actions at 60°/s. Participants were provided verbal encouragement during each contraction. The isokinetic extension resulting in the greatest peak torque value was used for analyses.

### Resistance training program for middle-aged participants

Middle-aged participants underwent supervised resistance training twice weekly for 10 weeks. Each session consisted of five exercises including leg/hip sled, leg extensions, lying leg curls, barbell bench press, and cable pull downs. For each exercise, participants performed 3 sets of 10-12 repetitions with at least 1 minute of rest in between sets. At the end of each set, participants were asked to rate the level of difficulty (0 = easy, 10 = hard). If values were below 7, weight was modestly added to increase effort. If values were 10, or the participant could not complete the set, weight was removed. Participants were encourage to be as truthful as possible when assessing difficulty. The intent of this training method was to consistently challenge participants where perceived exertion after each set was at a 7-9 rating. This method allowed us to ensure that training effort was maximized within each training session, and that the participants were successfully implementing progressive overload in an individualized fashion.

### Determination of muscle NAD^+^ and NADH concentrations

Muscle NAD+ and NADH concentrations were determined using a commercial assay (Abcam; Cambridge, MA, USA; catalog#: Ab65348) similar to previously published papers [53, 54]. Briefly, tissue was removed from -80°C storage and pulverized on a liquid-nitrogen-cooled stage. Powdered tissue (~20 mg) was placed in extraction buffer (400 μL) provided by the assay kit, and homogenized on ice using tight-fitting hard-plastic microtube pestles. Homogenates were then centrifuged at 15,000 g (2°C) for 5 minutes. Supernatants (10 μL) were assayed for total protein concentrations using a commercially-available bicinchoninic acid (BCA) kit (Thermo Fisher Scientific; Waltham, MA, USA) in order to normalize results, and the remainder of the supernatants (~3200 μL) were deproteinized through a 30 kD spin filter column at 15,000 g (2°C) for 60 minutes. For NADt (NAD^+^ and NADH), the spin column filtrate was collected and pipetted (50 μL) in duplicate on ice in a clear 96-well polystyrene microplate. Samples were incubated with a NAD cycling enzyme reagent provided by the kit to convert all NAD^+^ to NADH for 5 minutes at room temperature, and incubated with a color developer solution for 30 minutes at room temperature. For NADH-only determination, a portion of the spin column filtrate obtained above was pipetted into new 1.7 mL tubes and heated at 60°C for 30 minutes to decontaminate the sample of NAD^+^. Samples were cooled on ice, pipetted (50 μL) in duplicate on ice in a clear 96-well polystyrene microplate, and incubated with a color developer solution for 30 minutes at room temperature. After the 30-minute color development reaction, absorbances were read at OD450 using a plate reader (BioTek Synergy H1; Winooski, VT, USA), and concentrations (in pmol/50 μL) were extrapolated to a standard curve provided by the kit. Final concentrations were then normalized to μg of muscle protein provided by the BCA assay. Extreme care was taken during every step of the assay to keep samples or microplates on ice unless the kit instructions suggested performing steps at room temperature. Duplicate CV values for NADt and NADH averaged to be 7.1% and 9.9%, respectively. Due to tissue limitations, n=15/16 middle-aged participants and n=14/15 college-aged participants were assayed.

### Western blotting

Powdered muscle stored in foils were removed from -80ºC and placed on a liquid nitrogen-cooled ceramic mortar. Pre-crushed tissue (~20 mg) was weighed on a laboratory scale exhibiting a sensitivity of 0.0001 g (Mettler-Toledo; Columbus, OH, USA), and tissue was quickly placed in 200 μL lysis buffer (25 mM Tris, pH 7.2, 0.5% Triton X-100, 1x protease inhibitors). Samples were homogenized using tight-fitting hard-plastic microtube pestles on ice and centrifuged at 1,500 g for 10 minutes at 4°C. Supernatants were collected and placed in new 1.7 mL microtubes on ice for protein determination. Protein concentrations were determined using the commercially available BCA kit discussed above (Thermo Fisher Scientific). Samples were assayed in duplicate using a microplate assay protocol (20 μL of 5x diluted sample + 200 μL Reagent A + B). Afterwards, supernatants were prepared for Western blotting using 4x Laemmli buffer and distilled water (diH2O).

Samples (15 μL) were pipetted onto gradient SDS-polyacrylamide gels (4%–15% Criterion TGX Stain-free gels; Bio-Rad Laboratories; Hercules, CA, USA), and electrophoresis commenced at 180 V for 50 minutes. Following gel electrophoresis, proteins were transferred to pre-activated PVDF membranes (Bio-Rad Laboratories) for 2 hours at 200 mA. Gels were then Ponceau stained for 5 minutes, washed with diH2O for 1 minute, dried for 1 hour, and digitally imaged with a gel documentation system (ChemiDoc Touch; Bio-Rad Laboratories). Following Ponceau imaging, membranes re-activated in methanol, blocked with nonfat milk for 1 hour (5% w/v diluted in Tri-buffered saline with 0.1% Tween 20, or TBST), washed three times in TBST only (5 minutes per wash), and incubated for 48 hours with the following rabbit anti-[target] IgG antibodies (1:1000 v/v dilution in TBST): i) NAMPT (Abcam; catalog#: Ab45890), ii) SIRT1 (Cell Signaling Technologies; Danvers, MA, USA; catalog#: 9475), iii) SIRT3 (Cell Signaling Technologies; catalog#: 5490), and iv) acetylated lysine (Cell Signaling Technologies; catalog#: 9441). These antibodies have been extensively validated in prior literature: NAMPT [55, 56], SIRT1 [57, 58], SIRT3 [59, 60], acetylated lysine [61, 62]. Following primary antibody incubations, membranes were washed three times in TBST only (5 minutes per wash), and incubated for 1 hour with horseradish peroxidase-conjugated anti-rabbit IgG (Cell Signaling Technologies; catalog#: 7074). Membranes were then washed three times in TBST only (5 minutes per wash), developed using chemiluminescent substrate (Millipore; Burlington, MA, USA), and digitally imaged in a gel documentation system (ChemiDoc Touch; Bio-Rad Laboratories). Raw target band densities were obtained using associated software (Image Lab v6.0.1; Bio-Rad Laboratories), and these values were divided by Ponceau densities at 75-25 kD. These values were then divided by the grand mean of middle-aged participants at the Pre time point in order to obtain fold-difference values. All middle-aged participants were assayed; however, due to tissue limitations only n=14/15 college-aged participants were assayed.

### Determination of muscle global SIRT activity

Muscle global SIRT activity levels were determined in duplicate on supernatants obtained from muscle for Western blotting using an enzymatic assay (Abcam; catalog#: Ab156915). Prior to the assay, concentrations of supernatants were standardized to 2.5 μg/μL using diH2O, and 4 μL preparations were loaded in duplicate onto 96-well plates provided by the kit for enzymatic reactions as well as a no SIRT co-factor control reaction (NNC reaction, or an internal negative control reaction). Following execution of the assay per manufacturer’s recommendations, absorbances were read at OD450 using a plate reader (BioTek Synergy H1). NNC OD values were subtracted from enzymatic reaction OD values, and these values are presented in the results section as OD450 per 10 μg muscle protein. Duplicate CV values for all samples averaged to be 7.4%. All middle-aged participants were assayed; however, due to tissue limitations only n=14/15 college-aged participants were assayed.

### Determination of muscle citrate synthase activity in older participants

Muscle citrate synthase activity levels were determined in duplicate on supernatants obtained from muscle described in the Western blotting section; notably, these methods are similar to previous methods used by our laboratory [31, 33]. Muscle citrate synthase activity levels for each participant was used as a surrogate for mitochondrial density per the findings of Larsen et al. [63] suggesting citrate synthase activity strongly correlates with transmission electron micrograph images of intracellular space occupied by mitochondria (r=0.84, p<0.001). The assay principle is based on the reduction of 5,50-dithiobis(2- nitrobenzoic acid) (DTNB) at 412 nm (extinction coefficient 13.6 mmol/L/cm) coupled to the reduction of acetyl-CoA by the citrate synthase reaction in the presence of oxaloacetate. Briefly, 5 μg of skeletal muscle protein obtained from supernatants was added to a mixture composed of 0.125 mol/L Tris–HCl (pH 8.0), 0.03 mmol/L acetyl-CoA, and 0.1 mmol/L DTNB. All reactions occurred in 96-well plates, reactions were initiated by the addition of 5 μL of 50 mmol/L oxaloacetate per well, and the absorbance change was recorded for 60 seconds in a spectrophotometer (BioTek Synergy H1). The average CV values for all duplicates was 7.6%.

### Statistics

All statistical analyses were performed using SPSS v25.0 (IBM Corp, Armonk, NY, USA). Shapiro-Wilk tests on all dependent variables within the college-aged and middle-aged groups were performed prior to analysis to examine normality. In middle-aged participants, only Pre muscle NAD^+^ concentrations and Pre muscle NADH concentrations were non-normally distributed (p=0.007 and p=0.038, respectively). All dependent variables in college-aged participants presented Shapiro-Wilk p-values >0.100. Given that a majority of the data were normally distributed, we elected to perform parametric statistics on all data. For middle-aged participants, Pre versus Post data was analyzed using dependent samples t-tests. Gender comparisons were limited due to n-sizes for males being low. Notwithstanding, we also examined potential gender-training interactions using two-way repeated measures ANOVAs. The Pre and Post data from middle-aged participants was also compared to data from college-aged, trained participants using independent samples t-tests. Finally, Pearson correlations were performed between select dependent variables. All data are presented in figures and tables as means ± standard deviation (SD) values, and statistical significance was established as p<0.050.
